# Demographic and socioeconomic obstacles to access to malaria services for Myanmar migrants in Thailand

**DOI:** 10.1186/s12936-024-05066-y

**Published:** 2024-08-11

**Authors:** Piyarat Sripoorote, Yupaporn Wattanagoon, Nichakan Inthitanon, Pattamaporn Petchvijit, Waraporn Thongyod, Kyawt Mon Win, Ammarind Anantjitsupha, Naing Bo Bo Min, Nattawan Rachaphaew, Kritsana Suk-aum, Peeriya Watakulsin, Jetsumon Sattabongkot, Wang Nguitragool, Pyae Linn Aung

**Affiliations:** 1 Mahidol Vivax Research Unit, Faculty of Tropical Medicine, Mahidol University, Bangkok, Thailand; 2 Department of Clinical Tropical Medicine, Faculty of Tropical Medicine, Mahidol University, Bangkok, Thailand; 3 Center of Vector Borne Disease Control 2.3, Ministry of Public Health, Mae Sot, Thailand; 4 Office of Disease Prevention and Control 2, Ministry of Public Health, Phitsanulok, Thailand; 5 Department of Molecular Tropical Medicine and Genetics, Faculty of Tropical Medicine, Mahidol University, Bangkok, Thailand

**Keywords:** Barriers, Diagnostic, Malaria, Migrant, Prevention, Thailand–Myanmar border, Treatment

## Abstract

**Background:**

Typically mobile and vulnerable, migrants face significant barriers to access to routine malaria prevention, diagnostics and treatment, which leads to unchecked malaria transmission, particularly in border regions with a high population displacement. This study aimed to investigate the demographic and socioeconomic obstacles to access to malaria services among Myanmar migrants residing in the Thailand–Myanmar border areas.

**Methods:**

A cross-sectional study was conducted in early 2024 across three districts near the Thailand–Myanmar border. Quantitative data were collected from Myanmar migrants using standardized questionnaires through structured surveys. Data analysis included descriptive statistics and simple and multiple logistic regression models.

**Results:**

Out of 300 participants, approximately a quarter (27.3%) reported adequate access to comprehensive malaria services, including prevention, diagnostics, treatment and malaria-related health information. In multiple logistic regression models, factors associated with inadequate access included Myanmar migrants aged over 60 years (aOR: 7.63, 95% CI 1.74–20.58), accompanied by one to three family members (aOR: 3.33, 95% CI 1.06–8.45), earning monthly incomes below 3000 THB (aOR: 5.13, 95% CI 1.38–19.09) and 3000 to 6000 THB (aOR: 3.64, 95% CI 1.06–12.51), belonging to the Karen ethnicity (aOR: 2.13, 95% CI 1.02–3.84), with poor perception toward malaria (aOR: 2.03, 95% CI 1.03–4.01) and with poor preventive and health-seeking practices (aOR: 5.83, 95% CI 2.71–9.55).

**Conclusions:**

A significant proportion of Myanmar migrants encounter demographic and socioeconomic barriers to access to routine malaria services in Thailand. Tailored interventions are required to expand such access, including the recruitment of worksite health volunteers, strengthening the role of ethnic health organizations across the border and collaboration with private sector stakeholders (e.g. farm/company owners) to distribute preventive tools and ensure timely referral of suspected malaria cases to health facilities.

**Supplementary Information:**

The online version contains supplementary material available at 10.1186/s12936-024-05066-y.

## Background

Countries in the Greater Mekong Subregion (GMS) reported approximately 200,000 malaria cases in 2022 [1]. Among the six countries, Thailand holds a significant potential to achieve the countrywide elimination of malaria by 2030 following the successful elimination of malaria by China in June 2021 [2]. This progress is attributed to efforts that target the expansion of access to diagnostic and treatment services for malaria through community-based clinics and the deployment of village volunteers [3, 4]. Additionally, surveillance activities specific to the elimination of malaria, such as the 1–3–7 strategy of China initiated since 2016, have contributed to the continuous decline in malaria cases from 2012 to 2021 [1, 5]. In Thailand, however, the number of reported cases increased to nearly 17,000 in 2023 compared with 9989 in the previous year with approximately 42.0% classified as imported cases [1, 6]. Malaria remains endemic with ongoing transmission, particularly in provinces that border Myanmar [6–8]. For instance, Tak province near the Thailand–Myanmar border accounted for more than half of the reported malaria cases in the country, which experienced sporadic outbreaks [6, 9]. This increase can be attributed to factors such as the deterioration of the health system and the economic crises as a result of the political unrest in Myanmar that began in early 2021. Consequently, a significant population migration to neighbouring countries, notably Thailand, has occurred in search of safety and opportunities for livelihood. Given the persistent transmission in the border areas and the presence of competent vectors of malaria, including *Anopheles minimus*, *Anopheles maculatus*, *Anopheles annularis*, *Anopheles barbirostris* and *Anopheles dirus* [10–12], the influx of migrants could lead to the emergence of new cases of malaria. This potential scenario underscores the urgent need for intensified control measures for malaria tailored to the Myanmar migrants in these regions.

Access to malaria services, including prevention, diagnosis and treatment, is crucial for individuals residing in malaria-risk areas [13, 14]. However, providing access to malaria services for migrants is challenging due to their highly mobile nature and residence in geographically remote and hard-to-reach locations. Social and cultural barriers, such as isolation, discrimination and lack of integration into local communities, further compound the issue [2, 15]. Studies consistently demonstrate the poor utilization of long-lasting insecticide-treated nets (LLINs) among migrants [16–18]. Additionally, specific measures, such as insecticidal hammock nets and personal protective measures, such as using mosquito repellents and wearing long-sleeved clothes, may be required at workplaces. However, their utilization remains low due to factors such as lack of ownership and personal preferences [19, 20]. Furthermore, the ideal time for seeking care for malaria is within 24 h of the onset of fever. However, migrant populations exhibit poor malaria care-seeking behaviour, which increases the risk of severe symptoms and onward transmission within the range of host and mosquito travel [21]. To effectively interrupt localized transmission in Thailand, prioritising prevention, early diagnosis and standardized treatment is crucial. While Thailand provides regular active case detection in areas at low risk of malaria tailored for seasonal migrants, the effectiveness of this intervention remains minimal compared with passive case detection approaches despite being costly and low in prevalence [2, 22].

The International Organization for Migration estimates that Thailand hosts 4–5 million migrants with approximately 75% originating from Myanmar. Nearly half of these Myanmar migrants are believed to have undocumented status [23]. Frequently lacking official documentation to reside or work in Thailand, these migrants encounter numerous challenges, particularly access to healthcare services [24]. Financial constraints and perceived costs associated with healthcare services further compound the challenges they face [25]. Despite Thailand’s provision of free malaria diagnostic and treatment services regardless of immigration status (documented or undocumented), several barriers hinder access to these services, including language barriers and fears of legal repercussions [26]. Additionally, documented and undocumented migrants have the option to purchase health insurance in Thailand. However, the existing health and social protection regulations fail to ensure comprehensive access to services for migrants [13]. One report indicates that purchasing power toward health insurance schemes, including coverage for children, is notably low [27]. Moreover, legislation that addresses barriers to healthcare access for migrants, particularly regarding socioeconomic factors, remains inadequate [13]. In light of these challenges, this study aims to document the demographic and socioeconomic obstacles to access to malaria services for Myanmar migrants residing in the Thailand–Myanmar border areas.

## Methods

### Study design

This study employed a cross-sectional exploratory design among Myanmar migrants residing in Thailand regardless of immigration status. Data were collected using a quantitative approach at the community level from February to March 2024.

### Study settings

**Fig. 1:**
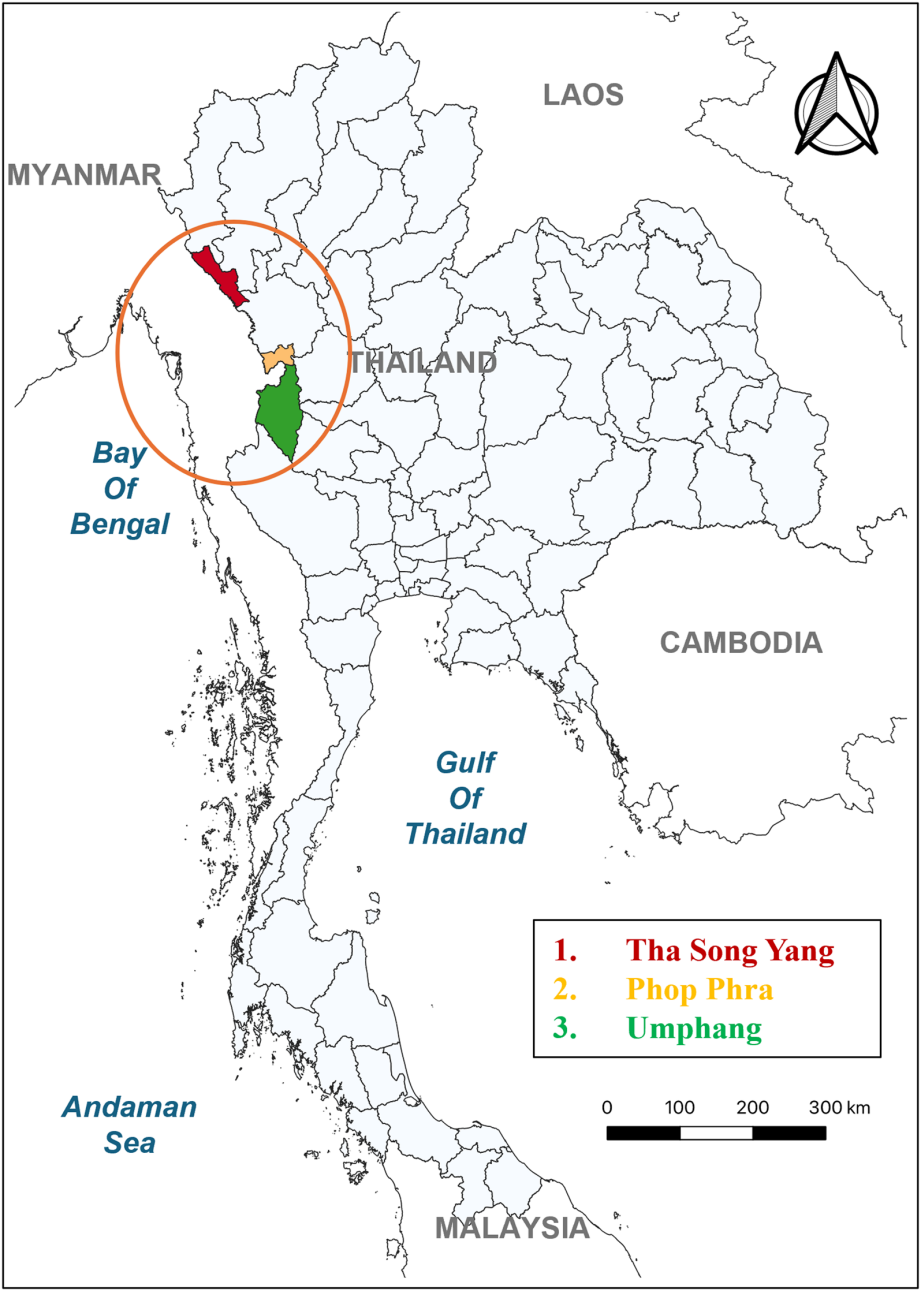
Locations of three study sites near the Thailand–Myanmar border

Tak province, which is situated near the Thailand–Myanmar border in northern Thailand, was selected as the study location due to its high malaria incidence and significant migrant population. From September 2022 to October 2023, Tak province reported more than 10,000 cases of malaria, which accounted for more than 60% of the total annual cases of malaria nationwide [1, 6]. Out of nine districts, the study selected three districts with the highest number of reported cases of malaria in 2023 as study sites. They are located adjacent to the Thailand–Myanmar border in which the Moei River serves as the natural boundary between the two countries (Fig. 1). The estimated population in these districts ranged from 34,000 to 95,000 with migrants from Myanmar constituting approximately 15.0% of the population. In 2023, each district reported approximately 1600 to 3000 cases of malaria [6].

Under the guidance of the Ministry of Public Health, the Department of Communicable Disease Control and the provincial public health office, a district vector-borne disease control centre along with healthcare staff from community-based malaria clinics and trained village malaria volunteers (VMVs) oversee the control activities for malaria in each district. On average, one VMV is assigned to malaria-related duties within 10–20 surrounding households. These duties include health education, referral of suspected cases of malaria to nearby malaria clinics and LLIN distribution. Malaria clinics provide free malaria diagnostics primarily through microscopy and treatment using first-line antimalarial medicines. LLIN distribution occurs biennially followed by annual continuous or targeted distribution that aims to reach underserved populations.

### Sample size and sampling

The sample size for this study was determined using the finite population proportion formula [28] with a reference value of 26.4%, which was derived from a previous study in Pailin, Cambodia [29], in which participants lacked knowledge of sources of LLINs. Accordingly, the required sample size for the current study was calculated as approximately 300 Myanmar migrants.

To achieve the required sample size, the study selected four to five villages from each district with the highest numbers of reported cases of malaria and a significant migrant population. With the assistance of the local health staff and VMVs from each district, the researchers conducted a thorough population census, including all migrants in each study area. Subsequently, it used systematic random sampling to reach the required sample size. If a randomized individual was absent at the time of the data collection, another individual was selected in their place.

Male or female individuals of Myanmar nationality aged more than 18 years and currently residing in Thailand were eligible for inclusion regardless of the duration of stay or immigration status (documented or undocumented). At the time of the survey, participants who did not agree to participate and those under the influence of drugs or alcohol, which could impair their ability to answer the questionnaire, were excluded based on observation.

### Data collection

Data were collected using a standardized quantitative questionnaire. The questionnaire was initially outlined in English then translated into Thai using the back-translation method conducted by two graduate research assistants. The researchers subsequently reviewed and finalized the translated version. Partially drawing from the malaria indicator survey of the World Health Organization (WHO) [30] and other relevant publications [13, 26, 31], the questionnaire comprised five parts, each with predefined choices from which the participants could select.

The first part aimed to explore socioeconomic characteristics, which covers aspects such as age, gender, number of visits to Thailand, duration of stay, occupation, level of education, number of accompanying family members, estimated monthly income in THB, ethnicity, language ability, lifetime malaria experience and travel time to reach the nearest health facility. The second part assessed their knowledge about malaria, which encompasses transmission, symptoms, diagnosis, treatment and prevention. The participants responded with ‘yes’ or ‘no’, and correct responses were given 1 point. Scores ranged from 0 to 27. The third part examined perceptions of malaria through 10 statements that addressed perspectives on causation, transmission, treatment and associated risks. Items were rated using a three-point Likert scale (*Agree*, *Neutral* and *Disagree*) [32] with total scores ranging from 10 to 30. The fourth section focused on preventive practices and health-seeking behaviours related to malaria and consisted of 30 statements related to fever-related illnesses, malaria history, health-seeking time and the use of LLINs and mosquito repellents. A correct response was given 1 point with total scores ranging from 0 to 30.

Lastly, the section on access to malaria services comprised three major questions on access to LLINs for prevention, diagnostic and treatment services for malaria and malaria-related health information. Access to LLINs was assessed by determining whether or not each migrant possessed any type of LLIN during their current stay in Thailand. Access to malaria diagnostics and treatment was defined by the knowledge of migrants of where they can obtain these services in Thailand. Access to malaria-related information referred to whether or not migrants received any information about malaria during their stay in Thailand from any source. The predefined answers under each question included positive and negative responses, and correct responses were given one point. If the migrants responded ‘no’ to any category, then they were considered to be without access to a particular category. For access to malaria diagnostics and treatment, however, responses were further validated through additional questions specific to each category even if the migrants indicated that they knew where to obtain these services. For example, migrants who initially reported knowledge on where to access treatment but later indicated they could only obtain treatment at inappropriate facilities, such as pharmacies, were categorized as without access. Adequate access was defined as full access to three malaria services (i.e. prevention, diagnosis and treatment and malaria related information), as recommended by the WHO [14]. The participants without access to at least one of these services were classified as having inadequate access.

About four to five VMVs, who have the ability to speak and understand multiple languages, including Thai, Karen and Burmese, were recruited as data collectors from each district. Prior to the survey, the research team organized a training session to familiarize the data collectors with the procedures of the study, consent processes, ethical considerations, quality assurance and practical aspects. Quantitative data were collected through structured surveys conducted by the trained data collectors in the Karen or Thai language according to the linguistic ability of the participant. Each data collector administered the researcher-led questionnaire at the village level and was occasionally supervised by the research team to ensure a smooth and accurate data collection process. Each survey session lasted 15 to 30 min.

### Data entry and analysis

The researchers coded and entered the data collected from the questionnaires into Microsoft Excel (Excel for Mac, version 16.84). The final raw dataset was then transferred to R (R Studio for Mac, version 2024.040 + 735) for further analysis. Sociodemographic characteristics and the itemized values for knowledge, perception, practices and access were descriptively presented using numbers and percentages. The total scores for each section were combined and illustrated using a box plot, including mean, median, minimum and maximum values. For the scores for knowledge, perception and practice, the participants were regrouped into good or poor categories based on whether their scores were below (< mean) or above (≥ mean) the mean. These categories were plotted on a bar chart along with 95% confidence intervals (CIs). Similarly, the total scores for each category under access to malaria services were presented using a bar chart.

Total access was regrouped as adequate or inadequate and served as the dependent variable. Meanwhile, the independent variables included in the models were categorized as follows: age groups (18–35, 36–60 and more than 60 years), gender (male or female), number of visits to Thailand (first time, 2–5 times or more than 5 times), duration of total stays in Thailand (less than 14 days, 14–60 days or more than 60 days), occupation (daily wage labour, agriculture, unemployed or others), level of education (primary or above), number of accompanying family members (alone, 1–3 or more than 3), monthly income (less than 3000 THB, 3000–6000 THB or more than 6000 THB), ethnicity (Karen or Burmese), language ability (ability to speak and understand Karen and Thai), lifetime malaria experience (never, 1–2 times, or more than 2 times), travel time to reach the nearest health facility (less than 15 min, 15–30 min or more than 30 min) and the categorization of knowledge, perception, preventive practices and health-seeking practices as good or poor. The study identified the factors related to inadequate access to malaria services using simple and multiple logistic regression models. All variables in the simple regression were retained in the multiple regression models regardless of significance to create a combined and balanced model that considers all constructed variables. Furthermore, to ascertain the strength of the associated factors, the study constructed additional multiple logistic regression models, including only the independent variables that exhibited significant associations in the simple regression model. Crude odds ratios, adjusted odds ratios (aORs), and 95% CIs were presented. A map of the study location was generated using QGIS for Mac (version 3.34.2-Prizren).

## Results

### Sociodemographic characteristics

The study recruited a total of 300 participants. Among them, more than half (55.3%) were aged 18–35 years, 52.7% were women, 56.7% were employed as daily wage labourers, 55.7% obtained a monthly income of less than 3000 THB and 60.0% lacked prior experience in contracting malaria. Additionally, approximately two-fifths (40.0%) visited Thailand for the first time, 41.4% stayed for more than 60 days and 40.3% resided more than 30 min away from the nearest health facilities. The majority (92.3%) reported their levels of education as below primary. Approximately two-thirds (77.7%) identified as Karen ethnicity, 66.7% were proficient in the Thai language and 72.3% were accompanied by one to three family members (Table 1).

**Table 1:** Sociodemographic characteristics of study participants (n = 300)

Characteristics	Number	%
Age (years)		
18 to 35	166	55.4
36 to 60	103	34.3
> 60	31	10.3
Gender		
Male	142	47.3
Female	158	52.7
Number of visits to Thailand		
First time	120	40.0
2 to 5 times	88	29.3
> 5 times	92	30.7
Duration of total stays in Thailand		
< 14 days	99	33.0
14 to 60 days	76	25.3
> 60 days	125	41.7
Occupation		
Unemployed	48	16.0
Agriculture	70	23.3
Daily wage labour	170	56.7
Others (Dependents, teachers, and students)	12	4.0
Education		
Primary school not completed	277	92.3
Primary school and above (Grade 5)	23	7.7
Numbers of accompanied family members		
Alone	26	8.7
1 to 3 members	217	72.3
> 3 members	57	19.0
Monthly income (THB^a^)		
< 3000	167	55.7
3000 to 6000	114	38.0
> 6000	19	6.3
Ethnicity		
Karen	233	77.7
Burmese	67	22.3
Language ability		
Able to speak and understand Thai	200	66.7
Able to speak and understand Karen	33	11.0
Able to speak and understand other than Thai and Karen	67	22.3
Lifetime malaria experience		
Never	180	60.0
1 to 2 times	96	32.0
> 2 times	24	8.0
Time to reach the nearest health facility		
< 15 min	99	33.0
15 to 30 min	80	26.7
> 30 min	121	40.3

^a^1 USD ~ 35 THB

### Knowledge about malaria

Table 2 summarizes the distribution of responses to the knowledge questions. A significant majority (72.6%) correctly identified malaria as transmitted through the bite of infected mosquitoes. However, nearly all respondents (95.7%) erroneously believed that malaria can be transmitted by drinking stagnant water, while only a minority (2.7%) mistakenly believed that consuming fruits, such as durian and banana, could transmit malaria. When asked about malaria symptoms, the majority (87.0%) correctly identified fever followed by chills and rigor (69.7%) and headache (61.7%). Moreover, more than two-thirds (69.3%) were aware that malaria can be diagnosed through blood testing. A number of respondents believed that they could receive malaria diagnosis at malaria clinics (29.7%) or through VMVs (18.3%), while a few (16.3%) believed they could self-diagnose based on present symptoms of the illness.

**Table 2:** Knowledge about malaria (n = 300)

Statements	Yes	
	Number	%
How can malaria be transmitted?		
Through the bite of infected mosquitoes	218	72.7
Eating durian/banana^a^	8	2.7
Drinking stagnant water^a^	287	95.7
Staying in the forest^a^	87	29.0
What are the symptoms of malaria?		
Fever	261	87.0
Chills and rigor	209	69.7
Sneezing and coughing^a^	17	5.7
Headache	185	61.7
Diarrhoea^a^	10	3.3
How can we diagnose malaria?		
By a blood test	208	69.3
By visiting a malaria clinic	89	29.7
By assessing symptoms ourselves^a^	49	16.3
By consulting village malaria volunteers	55	18.3
Cannot be diagnosed^a^	3	1.0
How can malaria be treated?		
By healthcare providers	49	16.3
By village malaria volunteers	95	31.7
By taking anti-malarial medicines	171	57.0
By taking traditional remedies^a^	22	7.3
It will automatically recover^a^	2	0.7
How can malaria be prevented?		
The use of bed nets	278	92.7
By using long-lasting insecticide-treated nets	29	9.7
By taking antimalarial medicines as chemoprophylaxis	13	4.3
Avoiding drinking stagnant water^a^	7	2.3
Not eating fruits such as banana, papaya and durian^a^	1	0.3
Applying mosquito repellent	48	16.0
Wearing long-sleeved clothes	28	9.3
Burning mosquito coils or rubbish^a^	22	7.3

^a^Negative answer

In terms of treatment, over half (57.0%) correctly identified taking anti-malarial medicines as treatment, while only a few (7.3%) believed that traditional remedies could cure malaria. Nearly all participants (92.7%) recognized the use of bed nets as a preventive measure against malaria. However, only a fraction considered personal protective measures, such as applying mosquito repellents (16.0%), using LLINs (9.7%) and wearing long-sleeved clothes (9.3%), as preventive measures. Furthermore, a few respondents mentioned burning mosquito coils or rubbish (7.3%) and avoiding drinking stagnant water (2.3%) as additional prevention measures against malaria.

### Perception toward malaria

The majority of the participants (73.7%) expressed the belief that malaria can be transmitted to Myanmar citizens while they are in Thailand. However, only approximately half (55.7%) perceived malaria as a potentially deadly disease. Additionally, slightly more than half (55.4%) believed that Myanmar migrants could access treatment services for malaria in Thailand in which many (68.7%) stated that the treatment for malaria in Thailand is free of charge. However, concerns emerged about the risk of being apprehended by officials when visiting malaria clinics (58.0%) as well as apprehension regarding the potential harmfulness of anti-malarial medicines (57.0%). Moreover, more than one-third (37.7%) were aware that Myanmar migrants also have access to LLINs in Thailand.

Approximately half (50.0%) continued to believe that malaria can be effectively treated using traditional medicines or drugs from a pharmacy, while slightly more than one-third (37.7%) expressed the misconception that a previous bout with malaria confers immunity to future infection. Additionally, a split opinion emerged regarding whether or not taking antimalarial medicines reduces the risk of disease transmission with 35.7% and 36.0% expressing belief in its efficacy and disagreement, respectively (Table 3).

**Table 3:** Perception toward malaria (n = 300)

Statements	Agree	Neutral	Disagree
	n (%)	n (%)	n (%)
Myanmar citizens cannot contract malaria in Thailand^a^	28 (9.3)	51 (17.0)	221 (73.7)
Malaria is a potentially deadly disease	167 (55.7)	94 (31.3)	39 (13.0)
Malaria can be treated with traditional medicines or drugs from a pharmacy^a^	150 (50.0)	53 (17.7)	97 (32.3)
Taking anti-malarial medicines reduces the risk of transmitting the disease to other people	107 (35.7)	85 (28.3)	108 (36.0)
Having suffered malaria once will invoke immunity that prevents future infection^a^	113 (37.7)	92 (30.6)	95 (31.7)
Myanmar citizens cannot access malaria treatment services in Thailand^a^	43 (14.3)	91 (30.3)	166 (55.4)
Myanmar migrants may not have access to long-lasting insecticide-treated nets in Thailand^a^	113 (37.7)	80 (26.6)	107 (35.7)
Visiting malaria clinics may pose a risk of being caught by officials^a^	54 (18.0)	72 (24.0)	174 (58.0)
Anti-malarial medicines are generally very harmful to us^a^	171 (57.0)	69 (23.0)	60 (20.0)
Malaria diagnostic and treatment services are free for us	206 (68.7)	53 (17.7)	41 (13.6)

^a^Negative statement

### Preventive practices and health seeking behaviours related to malaria

A total of 147 respondents (49.0%) reported an experience of fever during their stay in Thailand. Among them, a significant portion (41.7%) preferred seeking treatment at government hospitals, while approximately one-third (31.7%) opted to obtain medicine from a pharmacy, and more than one-fifth (21.0%) engaged in self-treatment. Furthermore, the majority (87.8%) delayed seeking treatment beyond 48 h after the onset of fever. Conversely, only a small fraction (12.7%) reported having contracted malaria. In terms of treatment preferences for malaria, approximately two-thirds (64.0%) favoured seeking treatment at government hospitals, while others sought assistance from VMVs (22.3%) or visited malaria clinics (12.7%).

Regarding the use of LLINs, more than half (55.7%) did not utilize them on the night prior to the survey. The common reasons included lack of access to nets (60.5%), financial constraints that prevented the purchase of nets (23.4%) and inability to set up LLINs in workplaces (21.0%). Furthermore, more than half (57.0%) never used mosquito repellents. Among them, the majority (67.3%) cited not owning repellents as the primary reason, while others mentioned financial constraints (43.4%) and concerns about the potential harmful effects of repellents such as skin allergies (15.2%) (Table 4).

**Table 4:** Malaria preventive practices and health-seeking behaviours (n = 300)

Statements	Yes	
	Number	%
Have you ever suffered from fever while staying in Thailand?	147	49.0
If you have a fever, how would you manage it?^a^		
Went to malaria clinics	22	7.3
Went to government hospitals	125	41.7
Went to private clinics	10	3.3
Went to village malaria volunteers	24	8.0
Took traditional remedies	31	10.3
Self-treatment	63	21.0
Took medicines form a pharmacy	95	31.7
It recovered by itself	6	2.0
When did you go to this facility after the onset of fever? (n = 147)		
Within 24 h	10	6.8
Within 24 to 48 h	8	5.4
More than 48 h	129	87.8
Have you ever suffered from malaria while staying in Thailand?	38	12.7
If you suffered malaria, how would you manage it?^a^		
Went to malaria clinics	38	12.7
Went to government hospitals	192	64.0
Went to private clinics	12	4.0
Went to village malaria volunteers	67	22.3
Took traditional remedies	13	4.3
Did you use bed nets or long-lasting insecticide-treated nets last night before the survey?		
If not, why? (n = 167)^a^		
Do not have any nets	101	60.5
Afraid of harmful effects of LLINs, such as skin allergies	4	2.4
Bed nets cannot be set up in the workplace	35	21.0
I do not need it as there are no mosquitoes	8	4.8
Cannot afford to buy	39	23.4
Thailand has no malaria	5	3.0
Have you ever used mosquito repellents?		
If not, why? (n = 171)^a^		
Do not have it	115	67.3
Afraid of harmful effects of repellents (E.g. skin allergies)	26	15.2
It is not effective in preventing mosquito bites	14	8.2
I do not need it as there are no mosquitoes	2	1.2
Cannot afford to buy	74	43.4
Thailand has no malaria	1	0.6

^a^in the survey responses, participants could have multiple answers. As a result, the cumulative total exceeded the overall number of participants

### Access to malaria services

Approximately two-thirds (59.7%) reported possessing LLINs: more than half of them (51.4%) received LLINs from ethnic health organizations (EHOs) or companies, whereas approximately one-third (33.0%) obtained them from their farm owners or employers. A similar proportion (28.5%) received them from the government or healthcare providers in Thailand. The majority of LLINs (68.2%) were acquired within one year in which more than two-fifths (40.8%) were obtained in the previous one to two years.

**Fig. 2:**
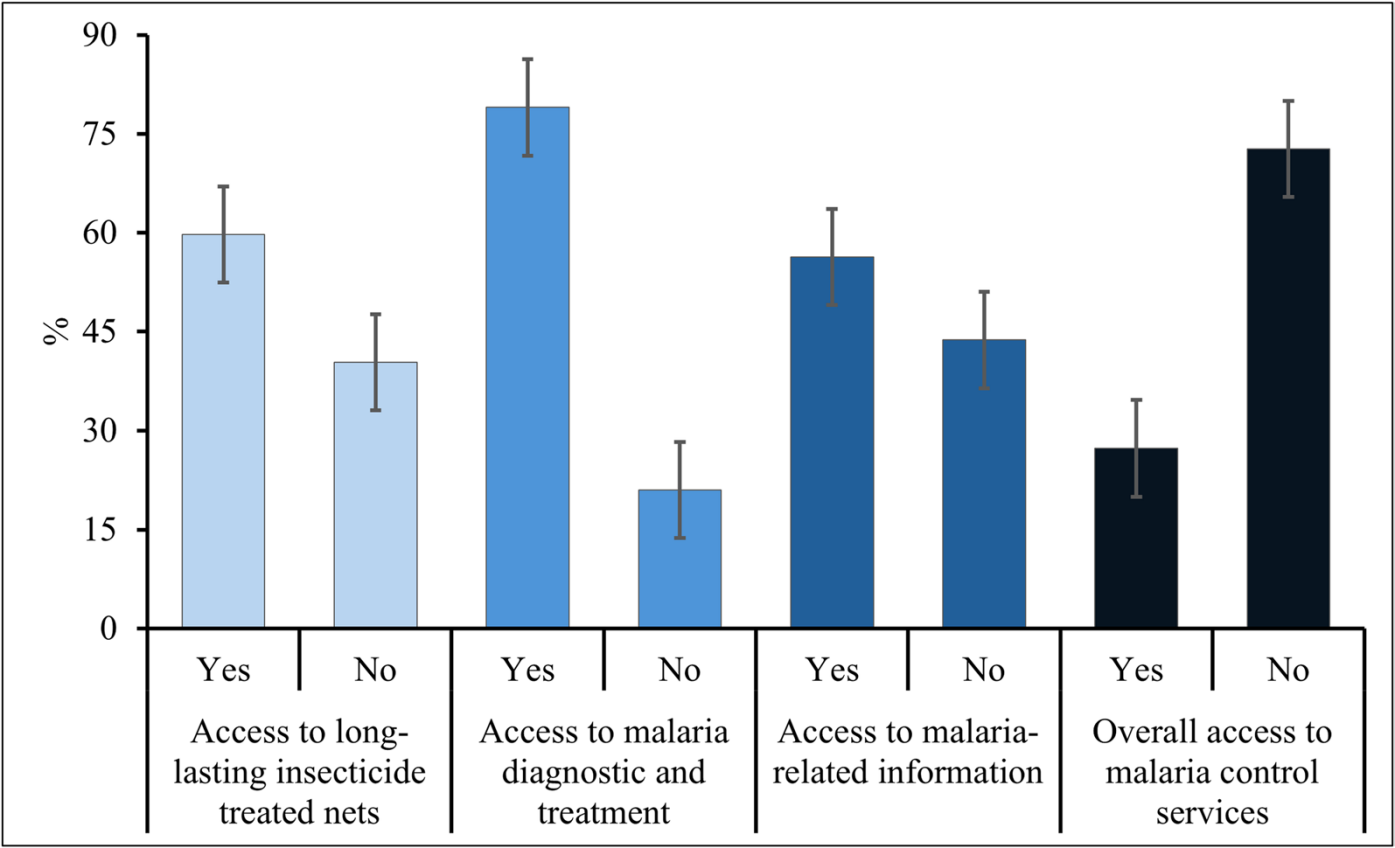
Overall access to malaria services

The majority (79.0%) were aware of where to access diagnostic and treatment services for malaria. Among them, more than half (51.9%) indicated governmental hospitals as their preferred choice followed by about two-thirds (42.2%) who mentioned malaria clinics. Additionally, a few participants mentioned VMVs (36.7%) and EHOs (15.6%) as sources of malaria services. More than half (56.3%) reported having received malaria-related information or education. The primary sources of this information were VMVs (50.3%), government hospitals (32.5%) and malaria clinics (21.3%) (Fig. 2 and Table 5).

**Table 5:** Access to malaria services (n = 300)

Statements	Yes	
	Number	%
Do you and your family possess long-lasting insecticide treated nets?		
If yes, where did you get them? (n = 179)^a^		
Took from Myanmar	16	8.9
Got from Thailand government/ healthcare providers	51	28.5
Bought from a shop	16	8.9
Received from farm owners or boss	59	33.0
Others (EHO and Company)	92	51.4
If yes, when did you get them? (n = 179)^a^		
Within 1 year	122	68.2
Within 2 years	73	40.8
More than 2 years	17	9.5
Do you know where you can receive malaria diagnostic and treatment services?		
If yes, where can you get them? (n = 237)^a^		
Malaria clinics	100	42.2
Village malaria volunteers	87	36.7
Pharmacy	14	5.9
Government hospital	123	51.9
Private clinics	2	0.8
Others (EHO)	37	15.6
Have you ever received malaria-related health information or education?		
If yes, where did you receive it? (n = 169)^a^		
Malaria clinics	36	21.3
Village malaria volunteers	85	50.3
Pharmacy	4	2.4
Government hospital	55	32.5
Private clinics	0	–
Friends or family members	26	15.4
Others (EHO, School health, Notice boards and NGOs)	18	10.7

EHO: Ethnic health organization; NGO: non-governmental organization

^a^in the survey responses, participants were permitted to select multiple options if they possessed more than one item. For instance, they could indicate ownership of more than one long-lasting insecticidal net, multiple repellents, various locations for episodes of fever, or receiving health information multiple times. As a result, the cumulative total exceeded the overall number of participants

Given the overall access to malaria services by balancing between these categories, the study found that only slightly more than a quarter (27.3%) of the participants had access to the three aspects of malaria services (Fig. 2).

### Total scores and grouping of malaria-related knowledge, perception and practice

**Fig. 3:**
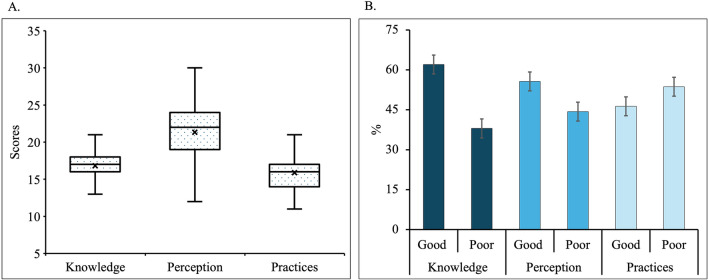
Malaria-related knowledge, perception, and practice scores (**A**) Combined scores of each. **B** Grouping according to mean scores

Figure 3A depicts the total scores for overall knowledge, perception and practices related to malaria. The participants achieved a mean knowledge score of 16.8, which ranged from 13 to 21 out of a possible 27 points. The mean perception score was 21.3 with minimum and maximum scores of 12 and 30, respectively, out of 30 points. In terms of practice scores, the mean was 15.89 with individual scores ranging from 11 to 21 out of 30 points.

After categorizing the groups into a good or poor category for knowledge, perception and practices using the mean scores as cut-off points, the study found that almost two-thirds (62.0%) belonged to the good knowledge group. Additionally, more than half of the participants obtained high scores for perception (55.7%) and practice (53.7%), respectively (Fig. 3B).

### Factors related to inadequate access to malaria services

Table 6 summarizes the factors related to inadequate access to malaria services. Out of the 300 participants, approximately three-quarters (72.7%) reported inadequate access. Descriptively, the highest rates of inadequate access according to characteristics were being female (77.2%), individuals aged more than 60 years (90.3%), visiting Thailand more than five times (85.9%), intending to stay for less than 14 days (82.8%) and engaging in agriculture-related occupation (87.1%). These rates also included individuals who had not completed primary education (73.6%), those who were accompanied by more than three family members (80.7%), identified as Karen ethnicity (76%), spoke and understood Thai (82.5%) and experienced malaria one to two times (81.3%). Lastly, those who lived more than 30 min away from the nearest health facilities (85.1%), exhibited good knowledge about malaria (73.7%), displayed poor perception toward malaria (73.7%) and poor preventive and health-seeking practices (84.5%) were also factors.

**Table 6:** Factors related to inadequate access to malaria services (n = 300)

Characteristics	No access (n = 218)	cOR (95%CI)	aOR (95%CI)
	n (row %)		
Age (years)			
18 to 35	108 (65.1)	Ref	Ref
36 to 60	82 (79.6)	2.10 (1.18–3.73)	1.50 (0.73–3.11)
> 60	28 (90.3)	5.01 (1.46–17.19)	7.63 (1.74–20.58)
Gender			
Male	96 (67.6)	Ref	Ref
Female	122 (77.2)	1.62 (0.97–2.71)	1.40 (0.69–2.82)
Number of visits to Thailand			
First time	78 (65.0)	Ref	Ref
2 to 5 times	61 (69.3)	1.22 (0.67–2.19)	1.70 (0.77–3.74)
> 5 times	79 (85.9)	2.27 (0.93–6.57)	1.17 (0.35–3.90)
Duration of total stays in Thailand			
< 14 days	82 (82.8)	1.15 (1.02–3.74)	0.96 (0.91–1.68)
14 to 60 days	47 (61.8)	0.66 (0.36–1.20)	0.46 (0.21–1.03)
> 60 days	89 (71.2)	Ref	Ref
Occupation			
Daily wage labour	112 (65.9)	Ref	Ref
Agriculture	61 (87.1)	2.51 (1.03–7.57)	1.70 (0.52–5.54)
Unemployed	38 (79.2)	1.97 (0.92–4.23)	0.38 (0.12–1.20)
Others (Dependents, teachers, and students)	7 (58.3)	0.73 (0.22–2.39)	0.26 (0.05–1.43)
Education			
Primary school not completed	204 (73.6)	1.80 (0.75–4.33)	2.89 (0.74–11.19)
Primary school and above (Grade 5)	14 (60.9)	Ref	Ref
Numbers of accompanied family members			
Alone	14 (53.8)	Ref	Ref
1 to 3 members	158 (72.8)	2.30 (1.00–5.25)	3.33 (1.06–8.45)
> 3 members	46 (80.7)	3.58 (1.30–9.88)	3.71 (0.91–15.19)
Monthly income (THB^a^)			
< 3000	137 (82.0)	4.11 (1.54–10.99)	5.13 (1.38–19.09)
3000 to 6000	71 (62.3)	1.49 (0.56–3.95)	3.64 (1.06–12.51)
> 6000	10 (52.6)	Ref	Ref
Ethnicity			
Karen	177 (76.0)	2.00 (1.13–3.57)	2.13 (1.02–3.84)
Burmese	41 (61.2)	Ref	Ref
Language ability			
Able to speak and understand Thai	165 (82.5)	3.38 (1.84–6.21)	15.18 (1.91–22.41)
Able to speak and understand Karen	14 (42.4)	0.53 (0.23–1.23)	1.71 (0.27–10.90)
Able to speak and understand other than Thai and Karen	39 (58.2)	Ref	Ref
Lifetime malaria experience			
Never	123 (68.3)	Ref	Ref
1 to 2 times	78 (81.3)	2.01 (1.10–3.66)	0.90 (0.39–2.08)
> 2 times	17 (70.8)	1.13 (0.44–2.87)	0.32 (0.09–1.21)
Time to reach the nearest health facility			
< 15 min	59 (59.6)	Ref	Ref
15 to 30 min	56 (70.0)	1.58 (0.85–2.95)	0.99 (0.43–2.30)
> 30 min	103 (85.1)	3.88 (2.04–7.37)	2.48 (0.94–6.54)
Knowledge about malaria			
Good	137 (73.7)	Ref	Ref
Poor	81 (71.1)	0.88 (0.52–1.48)	0.32 (0.15–0.69)
Perception toward malaria			
Good	120 (71.9)	Ref	Ref
Poor	98 (73.7)	1.10 (0.66–1.83)	2.03 (1.03–4.01)
Preventive practices and health seeking			
Good	82 (59.0)	Ref	Ref
Poor	136 (84.5)	3.78 (2.19–6.52)	5.83 (2.71–9.55)

^a^1 USD ~ 35 THB; cOR: crude odds ratio; aOR: adjusted odds ratio; CI: confidence interval; Knowledge, perception, and preventive practices regarding malaria were categorized into two groups: good (≥ mean) or poor (< mean), based on mean scores of 17 for knowledge, 21 for perception, and 16 for preventive practices

In multiple logistic regression models, individuals aged more than 60 years exhibited higher odds of acquiring inadequate access than did younger adults (aOR: 7.63, 95% CI 1.74–20.58). Similarly, individuals accompanied by one to three family members were prone to inadequate access compared with those travelling alone (aOR: 3.33, 95% CI 1.06–8.45). Those with monthly incomes less than 3000 THB (aOR: 5.13, 95% CI 1.38–19.09) and 3000 to 6000 THB (aOR: 3.64, 95% CI 1.06–12.51) were more likely to report inadequate access compared with those with incomes more than 6000 THB. For ethnicity, Karen individuals were more likely to obtain inadequate access than did the Burmese people (aOR: 2.13, 95% CI 1.02–3.84). The participants with poor perception toward malaria (aOR: 2.03, 95% CI 1.03–4.01) and poor preventive and health-seeking practices (aOR: 4.09, 95% CI 2.05–8.13) produced higher odds of having inadequate access to malaria services (Table 6).

Interestingly, individuals who could speak and understand Thai were more likely to experience inadequate access compared with those who could not (aOR: 15.18, 95% CI 1.91–22.41). Furthermore, the ability to speak and understand Thai remained significantly associated with poor access to malaria services in the alternative models (Additional file 1: Table S1). Additionally, those with good overall knowledge about malaria were more likely to have inadequate access to services than those with poor knowledge levels (aOR: 0.32, 95% CI 0.15–0.69), which is a relatively unexpected result. To further explore this finding, the scores for knowledge were classified into categories. The study observed that although many participants obtained high overall scores, they continued to display poor knowledge about certain aspects of malaria. The study further analysed each category of knowledge (transmission, symptoms, diagnosis, treatment and prevention) using the chi-squared test. The result indicated that inadequate access to services was significantly associated with poor knowledge of malaria diagnosis (*p* = 0.004) and prevention (*p* = 0.01) (Additional file 1: Table S2). Therefore, the per-protocol scoring method, which solely relies on the total score for knowledge alone, may not always be reliable.

## Discussion

The present study reveals that the majority of Myanmar migrants in Thailand lack access to essential services for malaria, which potentially perpetuates the ongoing transmission of malaria in the study areas. The lack of preventive measures, such as the ownership and use of LLINs, may sustain transmission chains among migrants and other residents. Additionally, the lack of access to diagnostic and treatment services can lead to unnecessary mortality and the emergence of symptomatic/asymptomatic carriers, which facilitates onward transmission. As such, interventions that are tailored to these individuals are imperative. While conducting active case detection through mobile clinics may be costly and less effective, especially among the highly mobile migrant population, adopting a passive case detection approach could prove advantageous [22]. This aspect could involve the expansion of diagnostic access potentially through the involvement of VMVs [33]. Furthermore, recruiting additional volunteers from among Myanmar migrants could enhance the effectiveness of such efforts. The current study also identifies the factors that contribute to the inadequate access to malaria services, including age, number of accompanying family members, ethnicity, language ability and knowledge, perception and practice related to malaria.

The present study identifies old age as a factor associated with inadequate access to malaria services among the Myanmar migrants. Typically, older individuals may not engage in agricultural-related employment due to retirement, which results in the lack of active income. Additionally, they may need to fulfil household responsibilities, such as caring for grandchildren or managing household chores, which can lead to a limited exposure to routine malaria information. Consequently, the majority of older people typically seek treatment for malaria from inappropriate providers and exhibit low levels of knowledge about malaria [34, 35]. In contrast, the working-age group, particularly those aged 18 to 35 years, frequently served as primary income earners and are more likely to prioritize their health. Similarly, low income was associated with inadequate access to malaria services [36]. Despite the availability of free diagnostic and treatment services for malaria provided by the Thai government [3], migrants may fear the associated costs or be concerned about their migration documentation status, which leads them to seek malaria treatment from inappropriate providers, such as pharmacies, or resorting to self-treatment [26, 37]. Given that the GMS is an epicentre for anti-malarial drug resistance, ensuring a complete and standardized treatment with recommended artemisinin combination therapies for migrant populations is crucial to the mitigation of the further spread of resistant strains [2]. Migrants arriving in Thailand with large family units also experienced inadequate access to malaria services. In such families, in which income levels are low, individuals may prioritize earning more income or attending to the needs of other family members over seeking malaria-related services. Previous studies document that individuals from large families typically exhibit poor health-seeking behaviours or knowledge about malaria, which places them at a higher risk of malaria transmission [38–40]. Therefore, older Myanmar migrants with limited income and large family sizes should be the targeted population for interventions that aim to improve access to essential malaria-related services.

Thailand shares its western border with Myanmar, particularly with the state of Kayin, in which the majority of the population are of Karen ethnicity. Historically, Karen people have temporarily and permanently migrated to Thailand primarily in search of job opportunities due to political unrest in Myanmar [41]. Consequently, they may display limited interest in prevention, diagnosis and treatment services for malaria, which reflects a poor perception toward malaria [42]. Given the geographical proximity and cultural similarities, including shared culinary traditions, between the two nations, many Karen people are similarly proficient in the Thai and Karen languages. However, despite this linguistic fluency, the majority of health promotional materials in Thailand are published in the local Thai language, such that only a few are available in Karen. Additionally, the educational system in Myanmar follows a standardized curriculum in the Burmese language [43], which may pose challenges for individuals of Karen ethnicity who are not proficient readers of Karen texts. Although they may understand spoken Thai, they may continue to struggle with reading the language, particularly when in relation to technical terms that pertain to malaria. Thus, linguistic ability plays a crucial role in the overall access to malaria services, including access to health information and care-seeking behaviours, among the migrant population [37, 44]. Furthermore, newly arrived short-term migrants may not yet be familiar with local malaria services regardless of language proficiency. For instance, a study conducted in Myanmar indicated that migrant groups with unstable living conditions experienced increased restricted access to information related to malaria [45]. Thus, efforts to improve access to malaria-related services should consider these linguistic barriers and provide appropriate materials and support tailored to the language proficiency levels of the target population.

The study also found that inadequate access to malaria services was associated with good knowledge, poor perception and poor preventive and health seeking practices related to malaria. Although these three factors are frequently interconnected with various aspects of a disease, including prevention and access to associated services, it does not guarantee a direct link between them. Good knowledge does not always translate into good practices, such that individuals with good knowledge may continue to be at risk of malaria due to other factors [46]. For instance, despite the good overall scores for knowledge produced by the migrants, their awareness regarding the crucial aspects of malaria services, such as diagnosis and prevention, remains limited. This gap contributes to the insufficient access to malaria services observed among the participants. Conversely, individuals with poor perception and practices may neglect diagnostic and prevention services, which further distances them from access to these vital resources. To address this issue and improve perception and practices, targeted health education interventions are crucial, especially for Myanmar migrants residing in Thailand. Despite their high levels of proficiency in the Thai language, reaching them through traditional mass gatherings is challenging due to their highly mobile nature [41]. Under such circumstances, contactless interventions that utilize mass media or mobile applications and the availability of behavioural change communication messages in multiple languages can be effective tools for the dissemination of important health information. However, one should acknowledge that poor preventive and health-seeking practices can occasionally serve as contributing factors, instead of obstacles, to overall access to malaria services. For instance, even if individuals know where to obtain diagnostic and treatment services, they may still not utilize them, which results in the lack of access to these services.

In this study, the overall utilization of LLINs on the night prior to the survey reached approximately 40.0%, which is primarily attributed to the lack of ownership and the logistical challenges associated with setting up nets at workplaces. This proportion is seemingly slightly lower than the reported LLIN usage rates of 53.1%, 52.0% and 68.3% among migrants in southern [47], central [17] and northern [16] Myanmar, respectively, but relatively higher than the rate of 39.0% among migrants in eastern Myanmar [21]. A targeted bed net distribution strategy that intends to achieve high coverage among these populations is imperative on both sides of the border, which facilitates the access of migrants to nets from Myanmar or enables them to obtain new ones in Thailand. For individuals who are unable to set up nets at their workplaces, the distribution of insecticidal hammock nets could be beneficial, especially given the low usage of mosquito repellents [46]. However, migrants typically exhibit reluctance in utilizing hammock nets [19, 31].

Despite the importance of early care-seeking behaviour, a significant portion of participants only sought a potential malaria diagnosis after 48 h since the onset of fever. Another study in this area found that the time to seek malaria treatment among patients with a confirmed diagnosis of malaria ranged from 1 to 26 days [48]. Timely care-seeking is critical in malaria cases to prevent progression to the severe stages of the disease and to mitigate onward transmission, particularly given the presence of competent malaria vectors such as *A. dirus* and *A. maculatus* [11, 49]. Given that migrants frequently enter Thailand for employment in agricultural farms or industrial companies, a viable approach could involve close collaboration with farm and company owners to facilitate the distribution of LLINs/insecticidal hammock nets or to ensure the timely referral of individuals suspected of having malaria to health facilities. Furthermore, implementing a tailored health insurance scheme for migrant workers could significantly enhance access to healthcare services at convenient health facilities, including diagnosis and treatment for malaria [50].

The study presents its strengths and limitations. This study is the first to explore the socioeconomic and demographic barriers to access to malaria services specifically among Myanmar migrants in Thailand, particularly in the context of increased population displacement after the military coup in Myanmar. Moreover, ethical consideration and the need to maintain the anonymity of the participants prevented the identification of immigration status, which may have led to an underrepresentation of the barriers faced by undocumented migrants. However, the active involvement of village-level malaria volunteers throughout the sampling and data collection processes may have helped capture information from a few of these populations. Data collected solely through questionnaires may not fully capture the complexity of the situation. For instance, assessing language ability was solely reliant on questionnaires, which may only partially reflect overall proficiency. Further research that uses qualitative methods to conduct an in-depth examination on the challenges faced by migrants in terms of access to malaria services could provide other valuable insights. Additionally, a number of Myanmar migrants may have returned to Myanmar during the study period due to their highly mobile nature, which could affect the representativeness of the results. Thus, the findings may reflect a snapshot of the situation at a specific point in time instead of fully representing all Myanmar migrants in the study location across years. The previous locations in which migrants resided prior to relocation in Thailand could significantly influence their overall knowledge, perception and practices related to malaria, which consequently impacts access to malaria services. For instance, individuals that originate from malaria-endemic regions with well-established control initiatives for malaria may have received more comprehensive health education and access to LLINs prior to migration.

## Conclusions

A significant proportion of Myanmar migrants in Thailand encounter demographic and socioeconomic barriers, which impede their access to routine malaria services. These barriers include older age, Karen ethnicity, low income, accompanied by multiple family members, poor malaria-related perception and poor preventive and health seeking practices. Tailored interventions, such as the expansion of diagnostic access through Myanmar worksite health volunteers and the provision of contactless health education via mobile applications using multi-language models, are necessary for addressing these obstacles and enhancing access to malaria services for this population. Potential strategies also include fostering collaboration with private sector farm/company owners and strengthening the roles of EHOs, while maintaining cross-border collaboration efforts. However, striking a balance between control initiatives for malaria with immigration laws and policies is crucial. Future research should conduct an in-depth investigation of the challenges faced by migrants and explore perspectives from healthcare providers potentially through qualitative research methods.

### Supplementary Information

**Additional file 1: Table S1. **Factors related to inadequate access to malaria services (n = 300). **Table S2. **Scores for each component of malaria-related knowledge and access to malaria services (n = 300).

**Additional file 2.** Quantitative questionnaire.

## Data Availability

All data generated or analysed for this study are included within the article. The de-identified raw dataset is available from the corresponding author upon reasonable request.

## Abbreviations

aOR: Adjusted odds ratio
CI: Confidence interval
cOR: Crude odds ratio
EHO: Ethnic health organization
GMS: Greater Mekong Subregion
LLINs: Long-lasting insecticidal nets
VMVs: Village malaria volunteers
WHO: World Health Organization
