# Supplementary material for: Demographic and socioeconomic obstacles to access to malaria services for Myanmar migrants in Thailand
Source: Malar J. 2024 Aug 11;23:239. doi: 10.1186/s12936-024-05066-y (PMC11318331; doi:10.1186/s12936-024-05066-y)
Supplement: Supplementary file 1 — Additional file 1: Table S1. Factors related to inadequate access to malaria services (n = 300). Table S2. Scores for each component of malaria-related knowledge and access to malaria services (n = 300). [file 12936_2024_5066_MOESM1_ESM.docx]

**Table S1**. Factors related to inadequate access to malaria services (n = 300).

| **Characteristics** | **No access**  (n = 218) | **cOR (95%CI)** | **aOR (95%CI)** |
| --- | --- | --- | --- |
|  | **n (row %)** |  |  |
| **Age (years)** | | | |
| 18 to 35 | 108 (65.1) | Ref. | Ref. |
| 36 to 60 | 82 (79.6) | 2.10 (1.18–3.73) | 1.85 (0.94–3.65) |
| > 60 | 28 (90.3) | 5.01 (1.46–17.19) | **6.80 (1.60**–**28.82)** |
| **Gender** | | | |
| Male | 96 (67.6) | Ref. |  |
| Female | 122 (77.2) | 1.62 (0.97–2.71) |  |
| **Number of visits to Thailand** | | | |
| First time | 78 (65.0) | Ref. |  |
| 2 to 5 times | 61 (69.3) | 1.22 (0.67–2.19) |  |
| > 5 times | 79 (85.9) | 2.27 (0.93–6.57) |  |
| **Duration of total stays in Thailand** | | | |
| < 14 days | 82 (82.8) | 1.15 (1.02–3.74) | 0.70 (0.26–1.86) |
| 14 to 60 days | 47 (61.8) | 0.66 (0.36–1.20) | 0.44 (0.21–1.02) |
| > 60 days | 89 (71.2) | Ref. | Ref. |
| **Occupation** | | | |
| Daily wage labour | 112 (65.9) | Ref. | Ref. |
| Agriculture | 61 (87.1) | 2.51 (1.03–7.57) | 1.82 (0.61–5.42) |
| Unemployed | 38 (79.2) | 1.97 (0.92–4.23) | 0.49 (0.17–1.46) |
| Others (Dependents, teachers, and students) | 7 (58.3) | 0.73 (0.22–2.39) | 0.36 (0.09–1.45) |
| **Education** | | | |
| Primary school not completed | 204 (73.6) | 1.80 (0.75–4.33) |  |
| Primary school and above (Grade 5) | 14 (60.9) | Ref. |  |
| **Numbers of accompanied family members** | | | |
| Alone | 14 (53.8) | Ref. | Ref. |
| 1 to 3 members | 158 (72.8) | 2.30 (1.00–5.25) | **2.50 (1.01**–**6.84)** |
| > 3 members | 46 (80.7) | 3.58 (1.30–9.88) | 3.26 (0.90–11.75) |
| **Monthly income (THB ^a^)** | | | |
| < 3,000 | 137 (82.0) | 4.11 (1.54–10.99) | **3.27 (1.01**–**11.18)** |
| 3,000 to 6,000 | 71 (62.3) | 1.49 (0.56–3.95) | 2.74 (0.85–8.82) |
| > 6,000 | 10 (52.6) | Ref. | Ref. |
| **Ethnicity** | | | |
| Karen | 177 (76.0) | 2.00 (1.13–3.57) | **2.22 (1.04**–**4.15)** |
| Burmese | 41 (61.2) | Ref. | Ref. |
| **Language ability** | | | |
| Able to speak and understand Thai | 165 (82.5) | 3.38 (1.84–6.21) | **5.95 (1.01**–**15.03)** |
| Able to speak and understand Karen | 14 (42.4) | 0.53 (0.23–1.23) | 1.59 (0.29–8.78) |
| Able to speak and understand other than Thai and Karen | 39 (58.2) | Ref. | Ref. |
| **Lifetime malaria experience** | | | |
| Never | 123 (68.3) | Ref. | Ref. |
| 1 to 2 times | 78 (81.3) | 2.01 (1.10–3.66) | 1.13 (0.53–2.40) |
| > 2 times | 17 (70.8) | 1.13 (0.44–2.87) | 0.35 (0.10–1.17) |
| **Time to reach the nearest health facility** | | | |
| < 15 minutes | 59 (59.6) | Ref. | Ref. |
| 15 to 30 minutes | 56 (70.0) | 1.58 (0.85–2.95) | 1.21 (0.56–2.63) |
| > 30 minutes | 103 (85.1) | 3.88 (2.04–7.37**)** | 2.75 (0.94–6.64) |
| **Knowledge about malaria** | | | |
| Good | 137 (73.7) | Ref. |  |
| Poor | 81 (71.1) | 0.88 (0.52–1.48) |  |
| **Perception toward malaria** | | | |
| Good | 120 (71.9) | Ref. |  |
| Poor | 98 (73.7) | 1.10 (0.66–1.83) |  |
| **Preventive practices and health seeking** | | | |
| Good | 82 (59.0) | Ref. | Ref. |
| Poor | 136 (84.5) | 3.78 (2.19–6.52) | **4.09 (2.05**–**8.13)** |

^a^ 1 USD ~ 35 THB; cOR: crude odds ratio; aOR: adjusted odds ratio; CI: confidence interval.

In this model, we included only independent variables that showed significant associations in simple regression into multiple logistic regression models to observe the extent of the association between good Thai language proficiency and poor access. Although the values were slightly reduced, the results did not change much. Therefore, we presented findings based on models that consider all constructed variables, regardless of their significance, to create a combined and balanced model.

Results showing significant associations are indicated by CI values in bold fonts.

**Table S2**. Scores for each component of malaria-related knowledge and access to malaria services (n = 300).

| **Knowledge level** | **Access** | | ***p*-value** |
| --- | --- | --- | --- |
|  | **Adequate**  **n (%)** | **Inadequate**  **n (%)** |  |
| **1. How can malaria be transmitted?** |  |  | 0.755 |
| Good | 45 (26.6) | 124 (73.4) |  |
| Poor | 37 (28.2) | 94 (71.8) |  |
| **2. What are the symptoms of malaria?** |  |  | 0.324 |
| Good | 28 (24.1) | 88 (75.9) |  |
| Poor | 54 (29.3) | 130 (70.7) |  |
| **3. How can we diagnose malaria?** |  |  | **0.004*** |
| Good | 25 (42.4) | 34 (57.6) |  |
| Poor | 57 (23.7) | 184 (76.3) |  |
| **4. How can malaria be treated?** |  |  | 0.98 |
| Good | 14 (27.5) | 37 (72.5) |  |
| Poor | 68 (27.3) | 181 (72.7) |  |
| **5. How can malaria be prevented?** |  |  | **0.01*** |
| Good | 31 (38.3) | 50 (61.7) |  |
| Poor | 51 (23.3) | 168 (76.7) |  |

*p*-value by chi-squared test; degree of freedom = 1; *significance at *p*-value < 0.05.

Two knowledge aspects regarding the ways to diagnose (*p* = 0.004) and prevent malaria (*p* = 0.01) were statistically significant with adequate access to malaria services.

The scores for each knowledge question were categorized into two groups: good (≥ mean) or poor (< mean), based on mean scores of 3 for question 1, 4 for question 2, and 3 for question 3, 3 for question 4, and 4 for question 5.
