# Supplementary material for: Demographic and socioeconomic obstacles to access to malaria services for Myanmar migrants in Thailand
Source: Malar J. 2024 Aug 11;23:239. doi: 10.1186/s12936-024-05066-y (PMC11318331; doi:10.1186/s12936-024-05066-y)
Supplement: Supplementary file 2 — Additional file 2. Quantitative questionnaire. [file 12936_2024_5066_MOESM2_ESM.pdf]

Quantitative questionnaire to assess malaria service access among Migrants

Part I. Socio-economic characteristics

**1.** Age	___________________years
**2.** Gender	Male / Female
**3.** How many times have you been in Thailand?	__________________times
**4.** Duration of current stay in Thailand	________month(s)_______day(s)
**5.** Occupation	1. Daily wage labour<br>2. Agriculture<br>3. Travelers<br>4. Dependent<br>5. Unemployed<br>6. Others_______________________
**6.** Normal working hours	From ______________to__________
**7.** Education completed	1. Primary school not completed<br>2. Primary school (grade 5)<br>3. Middle school (grade 9)<br>4. High school (grade 12)<br>5. College and above
**8.** How many family members come with you to Thailand?	_________________Members
**9.** Monthly family income (estimate)	____________________THB
**10.** Ethnicity	1. Karen<br>2. Mon<br>3. Burmese<br>4. Others_______________________
**11.** Language ability (select all the apply))	1. Karen<br>2. Burmese<br>3. Thai<br>4. Others_______________________
**12.** How many times have you experienced malaria in your life?	__________________times
**13.** Current residential subdistrict	1. Umphang<br>2. Phop Phra<br>3. Thasongyang
**14.** How long does it take to reach the nearest health facility?	1. Distance _____________ mile(s)<br>2. Time ________________ minutes

Part II. Knowledge about malaria

Knowledge question (select all the apply)		Yes	No
**1.** How can malaria be transmitted?			
	- Through the bite of infected mosquitoes		
	- Eating durian/ banana		
	- Drinking stagnant water		
	- Staying in the forest		
	- Others: ____		
**2.** What are the symptoms of malaria?			
	- Fever		
	- Chills and rigor		
	- Sneezing and coughing		
	- Headache		
	- Diarrhoea		
	- Others: ____		
**3.** How can we diagnose malaria?			
	- By a blood test		
	- By visiting a malaria clinic		
	- By assessing symptoms, ourselves		
	- By consulting village malaria volunteers		
	- Cannot be diagnosed		
	- Others: ____		
**4.** How can malaria be treated?			
	- By healthcare providers		
	- By village malaria volunteers		
	- By taking anti-malarial medicines		
	- By taking traditional remedies		
	- It will automatically recover		
	- Others____		
**5.** How can malaria be prevented?			
	- The use of bed nets		
	- By using long-lasting insecticide-treated nets		
	- By taking antimalarial medicines as chemoprophylaxis		
	- Avoiding drinking stagnant water		
	- Not eating fruits such as banana, papaya and durian		
	- Applying mosquito repellent		
	- Wearing long-sleeved clothes		
	- Burning mosquito coils or rubbish		
	- Others: ____		

Part III. Perception toward malaria

Statement	Agree	Neutral	Disagree
**1.** Myanmar citizens cannot contract malaria in Thailand.			
**2.** Malaria is a potentially deadly disease.			
**3.** Malaria can be treated with traditional medicines or drugs from a pharmacy.			
**4.** Taking antimalarial medicines reduces the risk of transmitting the disease to other people.			
**5.** Having suffered malaria once will invoke immunity that prevents future infection.			
**6.** Myanmar citizens cannot access malaria treatment services in Thailand.			
**7.** Myanmar migrants may not have access to long-lasting insecticide treated nets in Thailand.			
**8.** Visiting malaria clinics may pose a risk of being caught by officials.			
**9.** Anti-malarial medicines are generally very harmful to us.			
**10.** Malaria diagnostic and treatment services are free for us.			

Part IV. Access to malaria services

Accessibility question	Yes	No
**1.** Do you and your family possess long-lasting insecticide treated nets?		
1.1 If yes, where did you get them?		
- Took from Myanmar		
- Got from Thailand government/ healthcare providers		
- Bought from a shop		
- Received from farm owners or boss		
- Others: ____________________		
1.2 If yes, when did you get them?		
- Within 1 year		
- Within 2 years		
- More than 2 years		
**2.** Do you know where you can receive malaria diagnostic and treatment services?		
3.1 If yes, where can you get them?		
- Malaria clinics		
- Village malaria volunteers		
- Pharmacy		
- Government hospital		
- Private clinics		
- Others: ____________________		

**3.**	Have you ever received malaria-related health information or education?		
	4.1 If yes, where did you receive it?		
-	Malaria clinics		
-	Village malaria volunteers		
-	Pharmacy		
-	Government hospital		
-	Private clinics		
-	Friends or family members		
-	Others: ____		

V. Malaria preventive practices and health seeking behaviours

	**Preventive practice and health seeking behaviour**	**Yes**	**No**
**1.**	Have you ever suffered from fever while staying in Thailand?		
	If you have fever, how would you manage it?		
-	Went to malaria clinics		
-	Went to government hospitals		
-	Went to private clinics		
-	Went to village malaria volunteers		
-	Took traditional remedies		
-	Self-treatment		
-	Took medicines form a pharmacy		
-	It recovered by itself		
-	Others: ____		
When did you go to this facility after the onset of fever? ____________________Days			
**2.**	Have you ever suffered from malaria while staying in Thailand?		
	If you suffered malaria, how would you manage it?		
-	Went to malaria clinics		
-	Went to government hospitals		
-	Went to private clinics		
-	Went to village malaria volunteers		
-	Took traditional remedies		
-	Others: ____		
**3.**	Did you use bed nets or long-lasting insecticide-treated nets last night before the survey?		
	If no, why?		
-	Do not have any nets		
-	Afraid of harmful effects of LLINs, such as skin allergies		
-	Bed nets cannot be set up in the workplace		
-	I do not need it as there are no mosquitoes		
-	Cannot afford to buy		

- Thailand has no malaria		
- Others: ____		
**4.** Have you ever used mosquito repellents?		
If no, why?		
- Do not have it		
- Afraid of harmful effects of repellents, such as skin allergies		
- It is not effective in preventing mosquito bites		
- I do not need it as there are no mosquitoes		
- Cannot afford to buy		
- Thailand has no malaria		
- Others: ____		
